# Facile Synthesis of Carboxymethyl Cellulose Coated Core/Shell SiO_2_@Cu Nanoparticles and Their Antifungal Activity against *Phytophthora capsici*

**DOI:** 10.3390/polym13060888

**Published:** 2021-03-14

**Authors:** Nguyen Thi Thanh Hai, Nguyen Duc Cuong, Nguyen Tran Quyen, Nguyen Quoc Hien, Tran Thi Dieu Hien, Nguyen Thi Thanh Phung, Dao Khac Toan, Nguyen Thi Thu Huong, Dang Van Phu, Tran Thai Hoa

**Affiliations:** 1 Department of Chemistry, University of Sciences, Hue University, 77 Nguyen Hue Street, Hue City 530000, Vietnam; nguyenthanhhai@hueuni.edu.vn (N.T.T.H.); daokhactoancs80@gmail.com (D.K.T.); ntt.huong.sphoa@gmail.com (N.T.T.H.); 2 School of Hospitality and Tourism, Hue University, 22 Lam Hoang Street, Hue City 530000, Vietnam; 3 Pepper Research and Development Center, Pleiku City 600000, Vietnam; wasigl.quyen@ymail.com (N.T.Q.); wasigl.hien@ymail.com (T.T.D.H.); nguyenthanhphung11303096@gmail.com (N.T.T.P.); 4 Research and Development Center for Radiation Technology, Viet Nam Atomic Energy Institute, Ho Chi Minh City 700000, Vietnam; hien7240238@yahoo.com (N.Q.H.); phu659797@yahoo.com (D.V.P.); 5 Nguyen Binh Khiem High School, Chu Se District, Pleiku City 600000, Vietnam

**Keywords:** multicomponent SiO_2_@Cu@CMC nanoparticles, carboxymethyl cellulose, antimicrobial properties, *Phytophthora capsici*, fungicide, plant defense

## Abstract

Cu nanoparticles are a potential material for creating novel alternative antimicrobial products due to their unique antibacterial/antifungal properties, stability, dispersion, low cost and abundance as well as being economical and ecofriendly. In this work, carboxymethyl cellulose coated core/shell SiO_2_@Cu nanoparticles (NPs) were synthesized by a simple and effective chemical reduction process. The initial SiO_2_ NPs, which were prepared from rice husk ash, were coated by a copper ultrathin film using hydrazine and carboxymethyl cellulose (CMC) as reducing agent and stable agent, respectively. The core/shell SiO_2_@Cu nanoparticles with an average size of ~19 nm were surrounded by CMC. The results indicated that the SiO_2_@Cu@CMC suspension was a homogenous morphology with a spherical shape, regular dispersion and good stability. Furthermore, the multicomponent SiO_2_@Cu@CMC NPs showed good antifungal activity against *Phytophthora capsici* (*P. capsici*). The novel Cu NPs-based multicomponent suspension is a key compound in the development of new fungicides for the control of the *Phytophthora* disease.

## 1. Introduction

Copper compounds were first used in agriculture in 1761 [1], when it was discovered that a weak copper sulfate solution soaked in cereal seeds could inhibit the growth of fungal pathogens [2]. Nevertheless, it was not until the 1880s [2] that the farmers of the Bordeaux region, France, used a mixture of copper sulfate and lime as a fungicide against the downy mildew of grapes. This mixture was developed as an “accidental” invention, known as the Bordeaux mixture, and is still used commonly to prevent the spread of pathogenic fungi [3]. In 1885, Professor Millardet completed experiments using this mixture against downy mildew. Since then, the Bordeaux mixture has become known worldwide as a fungicide [4]. Copper’s antimicrobial activity is recognized in the world as well as being recorded by the US Environmental Protection Agency as the first solid antimicrobial material [2]. Nowadays, copper compound fungicides have become very important and thousands of tons of its compounds are used annually worldwide to prevent plant fungal diseases [5].

In recent years, the synthesis and use of novel antibacterial metal nanoparticles (NPs) has attracted a lot of interest due to the increase of drug resistance among microorganisms [6,7,8,9]. Among them, copper NPs with unique properties have demonstrated a significant enhancement of antibacterial activity compared with that of bulk copper metal because of their large ratio of surface area to volume [10]. Copper NPs possess a high biological activity, comparatively low cost and ecological safety, which could be considered as promising multifunctional antibacterial agents [11]. However, the preparation of stable copper NPs is a great challenge because Cu NPs are easily oxidized in air or aqueous media [12]. Several methods have been used to fabricate Cu NPs such as thermal decomposition [13,14], chemical reduction [15,16], thiol-induced reduction [17], reduction in micro-emulsions and reverse micelles [18,19], vapor deposition [20] and sono-electrochemical processes [21]. In most cases, the reduction of copper ions must be done in an inert atmosphere (specifically to clean O_2_ with N_2_ or Ar gas) [22]. It is well known that the reduction of copper ions into Cu NPs in a solution containing a stable agent can form a protective layer of NPs resulting in the NPs’ less exposure to air oxygen, leading to a reduction of the oxidation process. Furthermore, the decoration of Cu NPs on a substrate can improve their dispersion. Thus, the fabrication of multicomponent NPs constructed by the substrate nanoparticle inside followed by Cu NPs decorating on the substrate and outside as a protective polymer is an effective route for enhancing the stability of the properties of Cu NPs [22].

Carboxymethyl cellulose (CMC) is a soluble derivative of cellulose with carboxymethyl groups (–CH_2_–COOH) bound to some of the hydroxyl groups (–OH) of the glucopyranose monomers [23]. CMC contains a hydrophobic polysaccharide backbone and many hydrophilic carboxyl groups and hence shows amphiphilic characteristics. Due to its high solubility and viscosity in water, CMC is commonly used as a stable agent for NPs [24]. Amorphous silica (SiO_2_) with a biological activity is separated from rice husk ash. When activating silica in an alkaline environment, the silica nanoparticle surface forms –OH groups that can interact with Cu(II) ions. Cu NPs, formed after the chemical reduction of Cu(II) ions in a solution, decorate on the silica surface and increase the exposure surface of Cu NPs. The combination of advantages of CMC and silica NPs can synthesize a novel Cu NPs-based multicomponent suspension.

Black pepper (*Piper nigrum*) is one of main crops in Vietnam. Annually, the crop generates millions of US dollars to the Vietnam economy [25]. However, disease problems seriously affect disease problems seriously affect pepper cultivation leading to reduced yields or complete crop loss cultivation leading to reduced yields or complete crop loss [26,27]. One of the most devastating diseases of black pepper is foot rot disease caused by the pathogen *Phytophthora capsici* [28]. The disease infects black pepper with a reduction of about 2% of the total yield every year in Vietnam [25]. Controlling *Phytophthora capsici* (*P. capsici*) by chemical fungicides seems to be less effective because of the appearance of fungicide resistant germs. Moreover, the drawback of using chemical fungicides is that their residues leave serious, long-lasting effects on environmental and human health. Therefore, new approaches and strategies are being developed to control the fungi as well as enhance crop growth and productivity. In response to these increasing demands, the application of NPs has received a lot of attention due to its potential to protect plants and enhance plant growth.

In this paper, we report a simple method for the preparation of core/shell SiO_2_@Cu@CMC NPs. The [Cu(NH_3_)_4_]^2+^ complex was used as a starting material that was reduced by hydrazine to form a Cu ultrathin film covering on SiO_2_ NPs. CMC is both a stabilizer and a protective agent to limit oxidation of Cu nanolayers by oxygen. The obtained SiO_2_@Cu@CMC materials were tested against *P. capsici* fungi for their antifungal effect.

## 2. Materials and Methods

### 2.1. Materials

Amorphous silica NPs ~20 nm in diameter were prepared using rice husk ash as per the procedure of Sankar et al. [29] with a few modifications. The detail of the synthesis of the SiO_2_ NPs was described in our previous report [30]. Other chemicals, including copper sulphate pentahydrate (CuSO_4_.5H_2_O, 98%) and hydrazine mono hydrate (N_2_H_4_.H_2_O, 80%), were purchased from Merck (Darmstadt, Hesse, Germany). Carboxymethyl cellulose (CMC) and ammonium hydroxide (NH_4_OH, 25%) were purchased from Xilong Scientific Company (Shantou, Guangdong, China). The *P. capsici* was supplied by the Pepper Research and Development Centre, Western Highland Agriculture and Forestry Science Institute (Buon Ma Thuot City, Dak Lak, Vietnam).

### 2.2. Preparation of SiO_2_@Cu@CMC Nanocomposites

**Figure 1:**
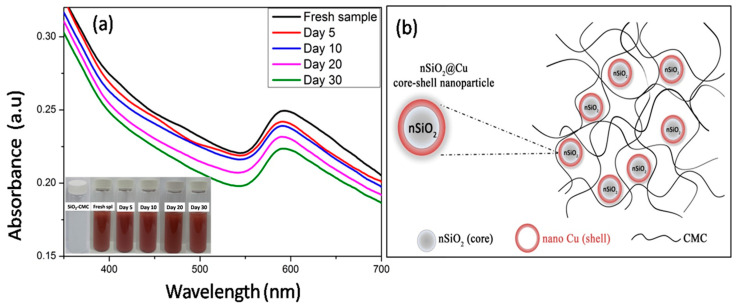
UV-vis absorption spectra of the as-prepared colloidal solutions as a function of time (**a**) and the scheme of SiO_2_@Cu@CMC nanoparticles (NPs) (**b**).

A total of 0.5 g of silica (SiO_2_) in 50 mL distilled water was mixed with 0.5 mL of 1 mM CuSO_4_ solution in a 200 mL beaker at room temperature. After that, 1 mL of 0.1 M NH_3_ solution was added the above mixture. The color of the mixture transferred from light blue into a dark blue color that indexed to the formation of the [Cu(NH_3_)_4_]^2+^ complex. After that, 100 mL of CMC 0.6% solution as a stable agent was added into the [Cu(NH_3_)_4_]^2+^ complex solution with vigorous stirring at 80 °C for 15 min and then 2 mL of 1 M N_2_H_4_ solution were added dropwise into this mixture with vigorous stirring at 80 °C for 30 min. The suspension transferred a dark blue color into a brown red color (inset in Figure 1a) indicating the formation of copper nanomaterials. After the centrifugation of the reaction mixture, we then collected and washed the precipitate with ethanol, then vacuum dried it to obtain the SiO_2_@Cu@CMC NPs.

### 2.3. Material Characterizations

The chemical structures of CMC, SiO_2_, SiO_2_@Cu and SiO_2_@Cu@CMC were analyzed by using a Fourier Transform Infrared Spectroscopy (FTIR) 8400S spectrometer (Shimadzu, Kyoto, Japan). The X-ray diffraction (XRD) pattern of SiO_2_ and SiO_2_@Cu NPs were recorded on an X-ray diffractometer, D8 Advance A25 (Brucker, Karlsruhe, Germany), in the scattering range two theta of 0–90° with a step rate of 0.25°/min. The particle sizes and morphologies of the SiO_2_ and SiO_2_@Cu samples were recorded using transmission electron microscopy (TEM) on a JEM1400 (JEOL, Tokyo, Japan). The elemental composition was determined by Energy-dispersive X-ray spectroscopy (EDX) analysis and HRTEM using JEOL 2100 and an EDX detector with XMax 80 T (Oxford). The UV-vis spectra were obtained using a Jasco V-550 UV-vis spectrophotometer within the range of 350–700 nm. The surface observation of SiO_2_@Cu@CMC samples was analyzed using electron dispersive X-ray analysis (EDX elemental mapping) (7593-H, Horiba, Japan).

### 2.4. Phytophthora capsici Preparation

*P. capsici* was isolated from soil samples taken from highly-infected pepper plantations in the Gia Lai province, Vietnam, following the method of Drenth and Sendall [31].

### 2.5. Antifungal Effect Test on Phytophthora capsici

Potato dextrose agar (PDA) was prepared and cooled to 50 °C. The SiO_2_@Cu@CMC NPs were then added separately to the medium to reach the following concentrations of the copper NPs: 0 ppm, 25 ppm, 50 ppm, 75 ppm, 100 ppm, 125 ppm and 150 ppm. The media were then poured into petri dishes (9 cm in diameter) with three dishes for each concentration. Mycelial discs (6 mm in diameter) of *P. capsici* were cut and put into the middle of the petri dishes. The diameters of the colonies were recorded every day.

*P. capsici* inhibition was measured by the formula I (%) = [(C–T)/C] × 100 [1], where I was *P. capsici* inhibition and T and C were mycelial disc diameters of treatment and control, respectively [32].

## 3. Results and Discussion

### 3.1. The Stability of the Suspension

To evaluate the stability of the SiO_2_@Cu@CMC suspension, the optical properties of the suspension containing the NPs were measured by UV-vis absorption according to the time and at room temperature. The UV-vis absorption of the fresh sample showed an absorption peak centered at about 600 nm, which assigned to the surface plasmon resonance of the metallic copper nanomaterials [33] decorating onto the surface of the silica NPs. The intensity of the absorption peak at ~600 nm decreased slightly, which may be assigned to the oxidation of the Cu NPs. However, the peak was still observed clearly after 30 days, demonstrating the SiO_2_@Cu@CMC suspension had a good stability. The stability of the sample may be related to the antioxidant protection provided by the protection of CMC. The possible formation mechanism of SiO_2_@Cu@CMC NPs is presented in Figure 1b.

### 3.2. Characteristics of the Obtained Materials

**Figure 2:**
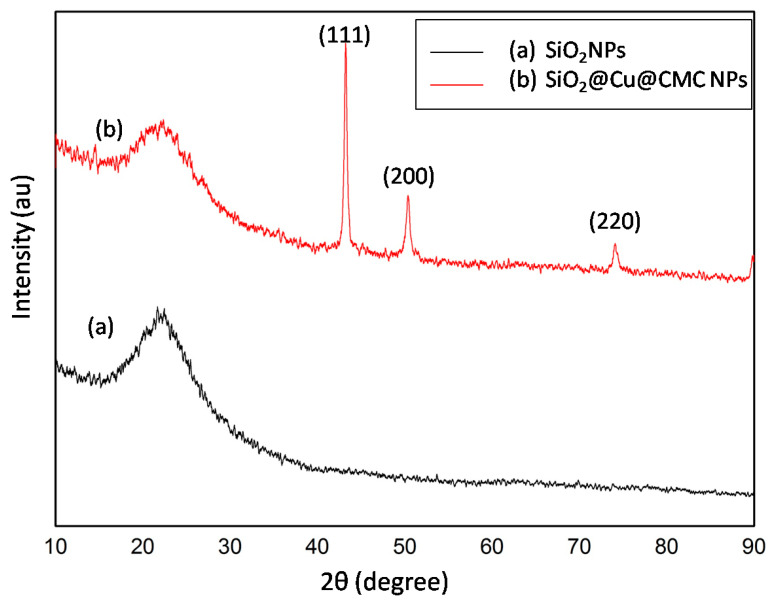
XRD patterns of the powder samples. XRD pattern of SiO_2_NPs (**a**) and XRD pattern of SiO_2_@Cu@CMC NPs (**b**).

The phase structure and the purity of the of the as-obtained SiO_2_@Cu@CMC and SiO_2_ NPs were examined by XRD as shown in Figure 2. The diffraction data presented in Figure 2a showed a broad peak at 2-theta range of 10 to 90 degrees, indexing to the amorphous SiO_2_ material [3]. Figure 2b shows the XRD patterns of the as-obtained SiO_2_@Cu@CMC NPs. All diffraction peaks indexed to (111), (200) and (220) planes at two theta ~43,21°, 50,25° and 74,15°, respectively, confirming the crystalline metallic phase Cu NPs with face centered cubic (FCC) structures (JCPDS Card No. 04-0838) [4]. The diffraction pattern of the sample also showed a peak for an amorphous structure of SiO_2_ NPs. The results showed that the pure crystalline phase Cu NPs were decorated on the surfaces of the silica nanoparticles.

**Figure 3:**
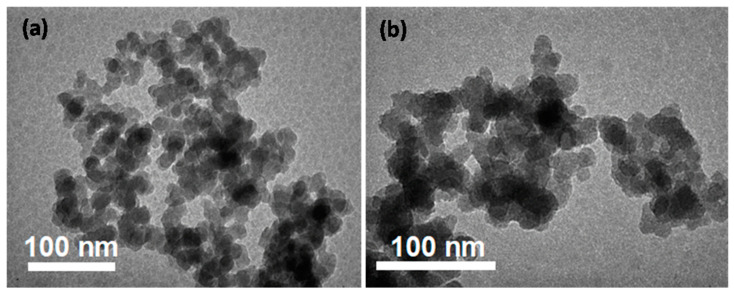
The TEM image of SiO_2_ NPs (**a**) and the TEM image of SiO_2_@Cu@CMC NPs (**b**).

The TEM images of SiO_2_ and SiO_2_@Cu@CMC NPs are shown in Figure 3. Figure 3 indicates that the SiO_2_ and SiO_2_@Cu@CMC NPs were fairly uniform in size. The particle shape of the SiO_2_ and SiO_2_@Cu NPs was nearly spherical with an average size of about ~19 nm. There was no significant change in the morphology and particle size between the SiO_2_ and SiO_2_@Cu@CMC materials. However, the SiO_2_@Cu@CMC tended to have more aggregation than that of the SiO_2_ NPs, which could be related to the CMC chains bonding together.

**Figure 4:**
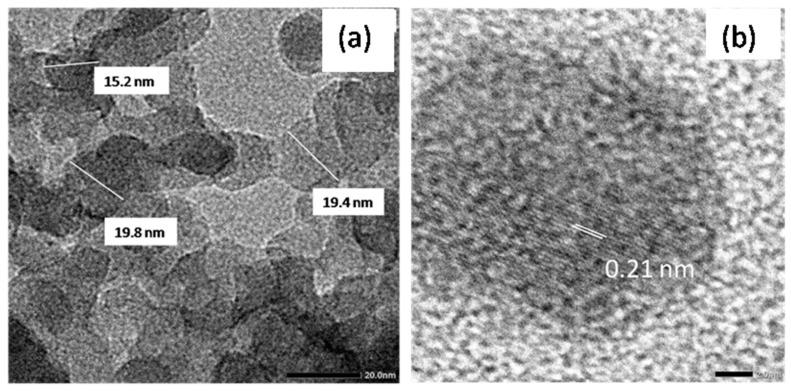
The high magnification TEM of the SiO_2_@Cu@CMC NPs (**a**) and HRTEM image of the SiO_2_@Cu@CMC NPs (**b**).

Further structural information of copper NPs was obtained from the HRTEM images shown in Figure 4a,b. From Figure 4a, the SiO_2_@Cu@CMC NPs were uniform in size and shape with their average size ~19 nm. From Figure 4b, the d-spacing of the lattice spacing between adjacent planes of 0.21 nm was clearly observed, which may correspond to the (111) planes of the FCC copper crystalline phase [34].

**Figure 5:**
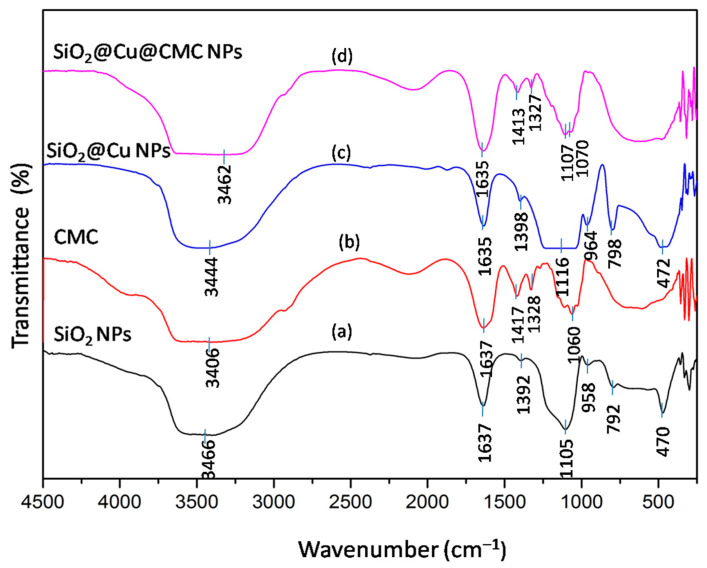
Infrared spectra of (**a**) SiO_2_ NPs, (**b**) CMC, (**c**) SiO_2_@Cu NPs and (**d**) SiO_2_@Cu@CMC NPs.

The FTIR spectra of CMC, SiO_2_, SiO_2_@Cu NPs and SiO_2_@Cu@CMC NPs are shown in Figure 5. Figure 5a is the FTIR of SiO_2_ NPs; the broad band near 3466 cm^−1^ corresponded to the O–H stretching vibration of the silanol group (Si–OH) condensation as well as the remaining absorbed water. A small peak appeared at 1637 cm^−1^ that was related to the bending vibration of the water molecules absorbed onto the surface of the silica particles [35]. Characteristic peaks appeared at 1105 cm^−1^, 792 cm^−1^ and 470 cm^−1^ that could be assigned to the asymmetrical stretching vibration of O–Si–O, the symmetrical stretching vibration of O–Si–O and the bending vibration of O–Si–O, respectively [36,37,38,39]. The peak of 1392 cm^−1^ related to the Si–O bond stretching and the band at 958 cm^−1^ indexed to the stretching vibrations of the silanol groups [35]. The FTIR spectrum of SiO_2_@Cu NPs in Figure 5c also exhibited typical vibrations like the FTIR spectrum of SiO_2_ NPs (Figure 5a); however, the peaks were shifted to the larger number of waves such as the peak of 3466 cm^−1^ to 3444 cm^−1^, the peak of 1392 cm^−1^ to 1398 cm^−1^ and the peak of 958 cm^−1^ to 964 cm^−1^. The results may confirm that Cu NPs were formed in the silica matrix [40]. Furthermore, Figure 5c shows no typical FTIR peaks for CuO at 400, 510 and 600 cm^−1^ [41].

The FTIR spectrum of CMC is shown in Figure 5b. It was obvious that the carboxymethyl and hydroxyl functional groups (OH) were found at wavelengths of 1637, 1417 and 1328 cm^−1^, respectively [42]. The strong absorption band at 1637 cm^−1^ confirmed the presence of COO−. The bands around 1417 and 1328 cm^−1^ were assigned to −CH_2_ scissoring and the hydroxyl group bending vibration, respectively. It could be seen that the broad absorption band at 3406 cm^−1^ was due to the stretching frequency of the hydroxyl group. The bands at 2931 and 1060 cm^−1^ were due to the C–H stretching vibration and −CH−O−CH_2_ stretching, respectively [42].

The FTIR spectrum of SiO_2_@Cu@CMC NPs is shown in Figure 5d. It also exhibited typical vibrations like the FTIR spectrum of SiO_2_@Cu NPs (Figure 5c). SiO_2_@Cu@CMC NPs showed C–C–C bending at 1413 cm^−1^, which demonstrated that CMC had coated the material.

**Figure 6:**
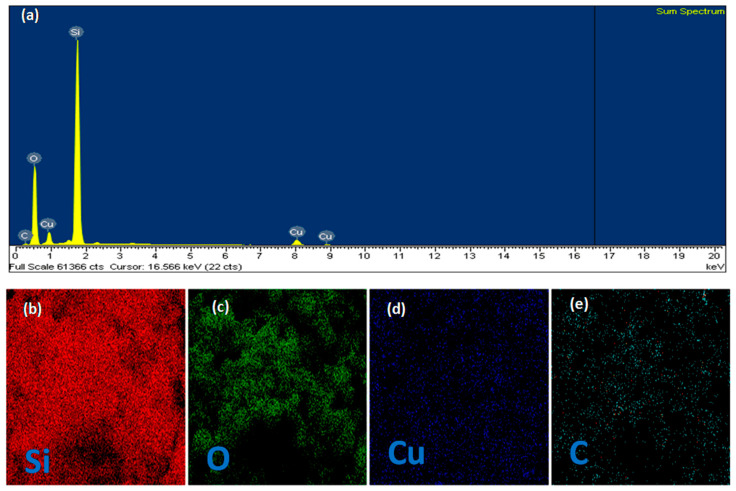
Typical EDX spectrum of SiO_2_@Cu@CMC NPs (**a**). EDX elemental mapping of Si (**b**), O (**c**), Cu (**d**) and C (**e**).

A more detailed analysis of the chemical composition of the surface of the SiO_2_@Cu@CMC NPs and the elemental mapping by scanning electron microscopy-energy dispersive X-ray (SEM-EDS) of the sample was characterized as shown in Figure 6. The SEM-EDS image in Figure 6a shows the presence of Si, Cu, O and C elements in the sample. The presence of silica (Figure 6b) and oxygen (Figure 6c) was revealed for the Si and O components of the spherical silica NPs. The elemental mapping image of Cu (Figure 6d) showed that the Cu element in the material was dispersed all over the silica substrate. Similarly, the elemental mapping image of C (Figure 6e) showed that C in CMC was evenly distributed and coated onto SiO_2_@Cu nanoparticles. These results demonstrate the formation of the multicomponent SiO_2_@Cu@CMC nanostructures.

### 3.3. Antifungal Effect Tests on Phytophthora capsici

**Figure 7:**
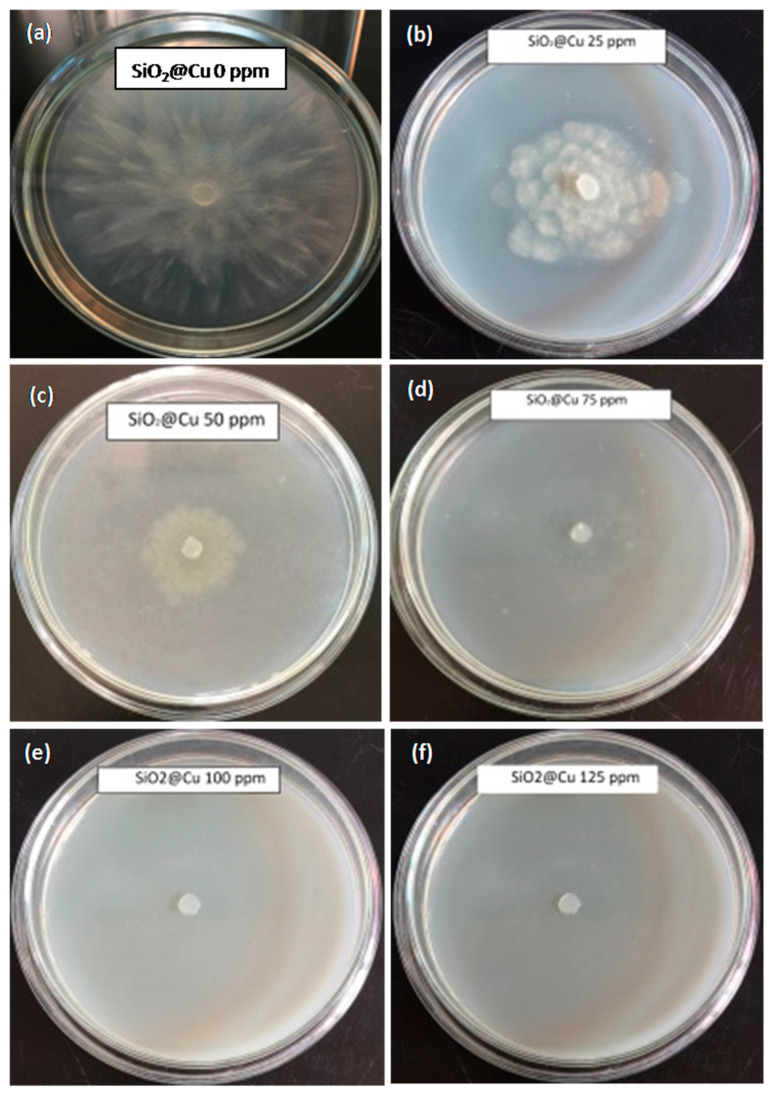
Mycelial growth of *P. capsici* after 72 h of incubation on potato dextrose agar (PDA) added by different concentrations of SiO_2_@Cu (**a**) 0 ppm, (**b**) 25 ppm, (**c**) 50 ppm, (**d**) 75 ppm, (**e**) 100 ppm, (**f**) 125 ppm.

Phytophthora foot rot of black pepper is caused by *P. capsici*, a soil-borne pathogen. This is a major disease of black pepper throughout Vietnam [43]. For a practical application, SiO_2_@Cu@CMC NPs were used for an antifungal effect test at six concentrations (0 ppm, 25 ppm, 50 ppm, 75 ppm, 100 ppm, 125 ppm). *P. capsici* inhibition was recorded after 24 h, 48 h and 72 h of incubation. The results are shown in Table 1 and Figure 7. In general, the inhibition effects increased after 24 h, 48 h and 72 h. After 24 h, the SiO_2_@Cu@CMC sample (a concentration of 25 ppm) showed fungus inhibition of around 49.11%. With an increase of SiO_2_@Cu@CMC concentrations from 25 to 125 ppm, the inhibiting effect to the fungus increased to 84.07%. After 48 h, there was an increase in the fungus inhibition up to 92.34% when the concentration of SiO_2_@Cu@CMC was of 75 ppm, 100 ppm and 125 ppm. At a SiO_2_@Cu@CMC concentration of 50 ppm, the sample showed 80.05% of an inhibiting effect. After 72 h, with the SiO_2_@Cu@CMC concentrations of 75 ppm, 100 ppm and 125 ppm these samples completely inhibited *P. capsici* growth up to 93.30% while there was a slight reduction at the SiO_2_@Cu@CMC concentration of 50 ppm with 73.37% of an inhibiting effect (Figure 7). The results indicated that the SiO_2_@Cu@CMC concentration of 75 ppm was the minimum inhibition concentration (MIC).

Different nanomaterials such as Ag_3_PO_4_ micro/nanocrystals [44], Ag NPs [45], Chitosan and chitosan-silver nanocomposites [46] have been used as effective antifungal agents to control *P. capsici*. Cu NPs can particularly be considered to be a promising fungicide against *P. capsici.* Pham et al. [47] reported that the colloidal solution of Cu NPs showed superiority in growth inhibition over *P. capsici.* The particle size of Cu NPs significantly affected their antifungal activity. The sample with smallest particle exhibited the highest growth inhibition activity because the NPs could easily penetrate the cell membranes through the surface. However, the antifungal activity of metallic Cu-based nanostructures against *P. capsici* has not been investigated fully. The antifungal mechanism of Cu NPs against the fungi might relate to the penetration of Cu NPs across the cell wall, which makes a change to the structure and function of the fungi cell thereby causing the death of the fungal microorganisms [48].

## 4. Conclusions

The core/shell SiO_2_@Cu@CMC NPs were successfully synthesized by a simple route. The as-synthesized SiO_2_@Cu@CMC NPs had a good solution stability with a particle size of ~19 nm. The multicomponent nanostructures constructed the SiO_2_ substrate nanoparticle inside followed by Cu NPs decorating on the silica substrate and outside as a CMC protective polymer. The SiO_2_@Cu@CMC exhibited a significant inhibition effect on *P. capsici*; the MIC was around 75 ppm. The results indicated that the SiO_2_@Cu@CMC nanomaterials were an ecofriendly candidate with excellent fungal resistance activities for use in agriculture to replace current toxic fungicides.

## Figures and Tables

**Table 1:** *P. capsici* inhibition by SiO_2_@Cu@CMC at different concentrations.

SiO_2_@Cu@CMC Concentration (ppm)	*P. capsici* Inhibition		
	24 h	48 h	72 h
0	0.00 ^g^	0.00 ^h^	0.00 ^h^
25	49.11 ^b^	54.78 ^c^	39.47 ^c^
50	84.07 ^a^	80.05 ^b^	73.37 ^b^
75	84.07 ^a^	92.34 ^a^	93.30 ^a^
100	84.07 ^a^	92.34 ^a^	93.30 ^a^
125	84.07 ^a^	92.34 ^a^	93.30 ^a^
P_0.05_	<0.0001	<0.0001	<0.0001

In the same column, different letters (e.g., a, b, c) show significant differences between treatments at P_0.05_.

## Data Availability

The data presented in this study are available on request from the corresponding author.
